# Integration of multi-omics technologies for molecular diagnosis in ataxia patients

**DOI:** 10.3389/fgene.2023.1304711

**Published:** 2024-01-04

**Authors:** Sebastien Audet, Valerie Triassi, Myriam Gelinas, Nab Legault-Cadieux, Vincent Ferraro, Antoine Duquette, Martine Tetreault

**Affiliations:** ^1^ University of Montreal Hospital Research Center (CRCHUM), Montreal, QC, Canada; ^2^ Department of Neurosciences, University of Montreal, Montreal, QC, Canada; ^3^ Department of Medicine, University of Montreal Hospital Centre (CHUM), Montreal, QC, Canada; ^4^ Neurology Service, Department of Medicine, André-Barbeau Movement Disorders Unit, University of Montreal Hospital (CHUM), Montreal, QC, Canada; ^5^ Genetic Service, Department of Medicine, University of Montreal Hospital (CHUM), Montreal, QC, Canada

**Keywords:** genomics, transcriptomics, ataxia, long-read sequencing (LRS), variant of uncertain significance (VUS), whole genome sequencing (WGS), RNA sequencing (RNAseq), multi-omics

## Abstract

**Background:** Episodic ataxias are rare neurological disorders characterized by recurring episodes of imbalance and coordination difficulties. Obtaining definitive molecular diagnoses poses challenges, as clinical presentation is highly heterogeneous, and literature on the underlying genetics is limited. While the advent of high-throughput sequencing technologies has significantly contributed to Mendelian disorders genetics, interpretation of variants of uncertain significance and other limitations inherent to individual methods still leaves many patients undiagnosed. This study aimed to investigate the utility of multi-omics for the identification and validation of molecular candidates in a cohort of complex cases of ataxia with episodic presentation.

**Methods:** Eight patients lacking molecular diagnosis despite extensive clinical examination were recruited following standard genetic testing. Whole genome and RNA sequencing were performed on samples isolated from peripheral blood mononuclear cells. Integration of expression and splicing data facilitated genomic variants prioritization. Subsequently, long-read sequencing played a crucial role in the validation of those candidate variants.

**Results:** Whole genome sequencing uncovered pathogenic variants in four genes (*SPG7*, *ATXN2*, *ELOVL4*, *PMPCB*). A missense and a nonsense variant, both previously reported as likely pathogenic, configured *in trans* in individual #1 (*SPG7*: c.2228T>C/p.I743T, c.1861C>T/p.Q621*). An *ATXN2* microsatellite expansion (CAG_32_) in another late-onset case. In two separate individuals, intronic variants near splice sites (*ELOVL4*: c.541 + 5G>A; *PMPCB*: c.1154 + 5G>C) were predicted to induce loss-of-function splicing, but had never been reported as disease-causing. Long-read sequencing confirmed the compound heterozygous variants configuration, repeat expansion length, as well as splicing landscape for those pathogenic variants. A potential genetic modifier of the *ATXN2* expansion was discovered in *ZFYVE26* (c.3022C>T/p.R1008*).

**Conclusion:** Despite failure to identify pathogenic variants through clinical genetic testing, the multi-omics approach enabled the molecular diagnosis in 50% of patients, also giving valuable insights for variant prioritization in remaining cases. The findings demonstrate the value of long-read sequencing for the validation of candidate variants in various scenarios. Our study demonstrates the effectiveness of leveraging complementary omics technologies to unravel the underlying genetics in patients with unresolved rare diseases such as ataxia. Molecular diagnoses not only hold significant promise in improving patient care management, but also alleviates the burden of diagnostic odysseys, more broadly enhancing quality of life.

## 1 Introduction

Ataxias encompass neurodegenerative disorders characterized by impaired coordination and balance, but also a remarkable clinical and genetic heterogeneity. With over 160 genes known to affect cerebellar functions, ataxia-related manifestations are found in a vast array of conditions such as spinocerebellar ataxias (SCAs), autosomal recessive cerebellar ataxias, episodic ataxias (EAs), spastic paraplegias, and mitochondrial disorders (Perlman et al., 1993; Synofzik et al., 2023). Furthermore, acquired ataxia can stem from non-genetic cerebellar damage, which can arise from autoimmunity, metabolic toxicity, vitamin deficiency, and infections (Nachbauer et al., 2015). These disorders exhibit a broad range of clinical features, most commonly unstable gait, dysarthria, visual disturbances, cerebellar atrophy, and muscle weakness. In addition to the age of onset and severity of clinical phenotypes being highly variable, disease presentation can extend beyond cerebellar functions, resulting in multi-systemic manifestations (Traschutz et al., 2021; Roberts et al., 2022). Part of the clinical heterogeneity and variable penetrance observed between ataxic individuals can be attributed to the diversity of potential pathogenic genomic alteration (Hersheson et al., 2012): single nucleotide polymorphisms, structural variants, copy number variants, repeat expansions, and splicing defects have all previously been reported throughout the extensive causative gene list. Genetic modifiers, while not fully understood, are also believed to play a role in the phenotypic spectrum of ataxia conditions (Rahit and Tarailo-Graovac, 2020). These factors underscore the need for a better understanding and classification of molecular causes, but also highlight the need for personalized clinical genetic testing methods.

EA is an intriguing category of ataxias that shares a lot of similarities to autosomal dominant SCAs, but distinguishes itself through the episodic nature of clinical ataxic manifestations, which can be triggered by various factors such as emotional stress or physical exertion (Choi and Choi, 2016). This atypical intermittence of symptoms, along with a frequent late-onset presentation, introduces additional layers of complexity to the diagnosis. Cases of progressive EA developing into chronic cerebellar ataxia also complicate the differential diagnosis (Giunti et al., 2020). There are officially eight subtypes of EA, although recent case reports suggest there could be up to eleven forms observed to this day (Hassan, 2023). In fact, pathogenic variants in *KCNA1* (EA1) and *CACNA1A* (EA2) are almost the only ones to be observed in multiple families, and represent the majority of EA cases (Sintas et al., 2017; Paulhus et al., 2020). In contrast, the other six subtypes were characterized mostly in single families (Escayg et al., 2000; Jen et al., 2005; Conroy et al., 2014), locating only three causative genes in *CACNB4* (EA5), *SLC1A3* (EA6), and *UBR4* (EA8). This is consistent with the notion that around 40% of patients who present late-onset phenotypes without an extensive family history do not obtain a molecular diagnosis despite a comprehensive evaluation (Perlman et al., 1993). Recent reports also suggest EA phenotypes can be caused by variants in genes traditionally associated with chronic disorders, and may often be overlooked or misdiagnosed in favor of more prevalent conditions (Hassan, 2023). Although recent advances have yielded new causative genes in *FGF14* (Schesny et al., 2019), *SCN2A* (Schwarz et al., 2016)*,* and *CACNA1G* (Gazulla et al., 2021), there is a clear need to investigate ataxia patients with episodic features to better define genetic etiology and improve clinical genetic screenings.

Whole genome sequencing (WGS) enables a complete overview of an individual’s genomic alterations, maximizing the chances of identifying novel pathogenic variants. The major limitation of WGS is that each individual carries millions of variants, with the majority of them still classified as variants of uncertain significance (VUS), comprising the majority of non-coding alterations as research on the matter is still sparse (Scacheri and Scacheri, 2015). This complicates variant prioritization, limiting the technology mostly to the exome regions, but still enabling a lower rate of inconclusive testing due to VUS than gene panels (Rehm et al., 2023). RNA sequencing (RNA-seq) on the other hand, offers valuable insights on the potential molecular effects of variants through expression and splicing data (Yepez et al., 2022). A multi-omics approach integrating genomic and transcriptomic sequencing provides a more comprehensive depiction of a patient’s genetic profile, thereby enhancing the potential for identifying previously unreported pathogenic variants in known or novel genes (Lunke et al., 2023). The latter does require further functional validation, which is where the versatility of long-read sequencing (LRS) is highly interesting. It is been shown that the technology allows for precise characterization of transcriptomic landscapes (Mezreani et al., 2022), large structural variants (Miller et al., 2022), and even repeat expansion (Pellerin et al., 2023). Improvements in the capacity to quickly identify underlying causes of genetic disorders offers many benefits to patients, including better care management and treatment options following the molecular diagnosis. Furthermore, with the continuous advancements in personalized genetic therapy tools, such as antisense oligonucleotides, gaining a comprehensive understanding of the genetic variations at play becomes increasingly crucial. Molecular diagnoses now present a promising perspective for developing effective treatments tailored to these individuals (Kulkarni et al., 2021).

In this study, we describe a multi-omics approach to uncover potential disease-causing variants in late-onset ataxia patients with episodic features lacking molecular diagnoses despite exhaustive clinical and genetic evaluation. We combined WGS, RNA-seq as well as LRS to identify variants and validate segregation of alleles, splicing defects and repeat expansions. Our results highlight the need to broaden genetic testing in unsolved patients with primary EA or EA-like phenotypes.

## 2 Materials and methods

### 2.1 Participants and clinical investigation

The cohort is comprised of 7 probands from different families, with a single affected family member in patient #6 (related to patient #5). All participants were referred for episodes of ataxia and had a comprehensive clinical evaluation in July 2019 at the time of specimen collection. Neurological and general examination were performed and the Inventory of Non-Ataxia Signs (INAS) as well as the Scale for the Assessment and Rating of Ataxia (SARA) were recorded (Schmitz-Hubsch et al., 2006; Jacobi et al., 2013). Medical files were reviewed to document previous genetic panels and imaging studies. Acquired causes of ataxia were investigated through systematic questioning as well as laboratory tests including vitamin levels, and autoimmune and paraneoplastic markers. The Movement Disorders Society criteria were used to rule out multi-system atrophy (Wenning et al., 2022). All data was recorded in the REDCap database software developed at Vanderbilt University (Harris et al., 2019). Participants were recruited for further sequencing in light of a suspected complex ataxia without a molecular diagnosis. The study was approved by the hospital’s ethics committee, and recruited participants signed an informed consent form authorizing genetic analysis for research purposes.

### 2.2 Sample preparation

Participants had blood samples drawn as part of their clinical work-up. Peripheral blood mononuclear cells were isolated using a standard Ficoll-paque centrifugation protocol, and 2.5 million cells pellets were frozen at −80 °C prior to their extraction with a column purification kit (Norgen Biotek™). DNA and RNA were quantified using the QuBit system (Thermo Fisher Scientific™) with high-sensitivity kits prior to storage at recommended temperatures for sequencing and validation experiments.

### 2.3 Whole genome sequencing

DNA samples were diluted in TE buffer and loaded onto Twin-Tec sequencing plate (Eppendorf™) for quality assessment, library preparation, and sequencing from Centre d’expertise et de services Génome Québec (CESGQ). The shotgun PCR-free genomic library was prepared using dual-index adapters from Integrative DNA technologies™. Illumina™ NovaSeq 6000 S4 sequencers provided at least 70 million paired-end reads (150bp) coverage for each sample.

### 2.4 RNA sequencing

RNA samples were diluted with molecular grade water in 1.5 mL tubes for transport on dry ice to CESGQ. Quality control was performed with Agilent™ Bioanalyzer 2100 to ensure an integrity value (RIN) greater than 8.0. Library preparation was performed by PolyA capture method with next-dual adapters from New England Biolabs™. Illumina™ NovaSeq 6000 S4 technology generated a minimum of 50 million paired-end reads (100bp) coverage for each sample.

### 2.5 Multi-omics variant analysis

For both sequencing data, quality was assessed with MultiQC and FastQ files were processed with trimmomatic prior to their alignment on the hg19 reference genome (Bolger et al., 2014; Ewels et al., 2016). Whole genome data was aligned using BWA, while STAR was used for RNA-seq (Li and Durbin, 2009; Dobin et al., 2013). Variant calling was performed using GATK best practices recommendations for both datasets (McKenna et al., 2010), and annotations were added using ANNOVAR (Wang et al., 2010). Variants with a minor allele frequency greater than 1% in gnomAD were excluded. Multiple tools supplemented the genomic investigation: CNVkit and LUMPY were used to cover calling of structural variants of all sizes (Layer et al., 2014; Talevich et al., 2016), ExpansionHunter and STRetch predicted expansions of microsatellites in known pathogenic regions as well as genome-wide (Dashnow et al., 2018; Dolzhenko et al., 2020), and SpliceAI predicted variants impacting splicing in coding and non-coding regions (Jaganathan et al., 2019). Quantification of RNA expression utilizing both Salmon and featureCount (Liao et al., 2014; Patro et al., 2017), as well as differential expression analysis using DESeq2 (Love et al., 2014), served for gene expression examination. Non-canonical splicing junction calling with rMATS also helped with variant interpretation (Shen et al., 2014). Custom scripts were used to further filter and process the data, in respect to standardized best practices for clinical variant prioritization, and utilized *in silico* predictions such as the Combined Annotation-Dependent Depletion (CADD ≥10) scores (Rentzsch et al., 2019). Relevance of the variants to phenotypes was assessed with literature reports from GeneCards (Stelzer et al., 2016), The Human Protein Atlas (Uhlen et al., 2019), Uniprot (UniProt, 2023), gnomAD (Chen et al., 2022), OMIM (Hamosh et al., 2005), Mutation Taster (Steinhaus et al., 2021) and ClinVar (Landrum et al., 2018). Overall, non-synonymous low-frequency variants predicted to be damaging, putative splicing variants, coding indels, CNVs and repeat expansion in genes directly or indirectly associated with ataxias were prioritized.

### 2.6 Quantitative polymerase chain reaction

RNA from cohort samples was obtained as described in the sample preparation section, while healthy controls had previously been extracted with a TRIzol (Thermo Fisher Scientific™) protocol. Hence, both were used separately to control for batch effect. RNA reverse transcription was carried out using SuperScript Vilo mix (Thermo Fisher Scientific™). 10ng of cDNA was loaded into a 384-well plate (Thermo Fisher Scientific™) along with a FAM-MGB Taqman probe for a gene of interest and an optimized fluorescence master mix (Thermo Fisher Scientific™). Probes for *ELOVL4*, *PMPCB*, and *ZFYVE26* (Hs00224122_m1; Hs01012489_m1; Hs00188704_m1) were normalized using the housekeeping gene *ACTB* (Hs01060665_g1). Expression levels were assessed through relative ΔΔCT quantifications obtained from Applied Biosystems™ QuantStudio 6/7 systems.

### 2.7 Long-read sequencing

Libraries were prepared from either genomic DNA or reverse transcribed cDNA, and were PCR amplified (30 cycles) using the LongAmp 2X Taq Polymerase (New England Biolabs™). Primers designed with primer3 (Untergasser et al., 2012) targeted one of *SPG7*, *ELOVL4*, *PMPCB*, or *ATXN2* (Supplementary Table S1). Libraries end prepping utilized NEBNext Ultra II End Repair/dA-Tailing Module (New England Biolabs™; Cat. E7546) to allow barcode ligation (kit SQK-NBD112.24) using the Blunt/TA ligase (New England Biolabs™; Cat. M0367) prior to Nanopore™ sequencing adapters ligation with the NEBNext Quick Ligation Module (New England Biolabs™; Cat. E6056). Purification with included AMPure XP beads is performed between each step for optimal results, with the last clean-up utilizing a size-specific retention buffer from Oxford Nanopore™. Multiplexed libraries were quantified using the QuBit4 fluorometric system for optimal flow cell loading and maximized sequencing over a 72-h period. Generated data were processed with Guppy for high-accuracy base calling and demultiplexing (Wick et al., 2019), minimap2 for alignment to the hg19 reference genome (Li, 2018), and PycoQC for quality assessment (Leger and Leonardi, 2019). Transcriptomic results were then generated using FLAIR as recommended by the authors (Tang et al., 2020). The “align” and “correct” functions used hg19 reference genome and Gencode v19 annotations (Frankish et al., 2021), “collapse” was set in best-only mode with a threshold of 500 supporting reads, and “quantify” utilized the–trust-end parameters since adapters were removed during demultiplexing. Targeted repeat expansion was quantified using tandem-genotypes tool with standard parameters (Mitsuhashi et al., 2019). The resulting data were used to construct the customized plots presented in the article.

## 3 Results

### 3.1 Individual #1 *SPG7*

**FIGURE 1:**
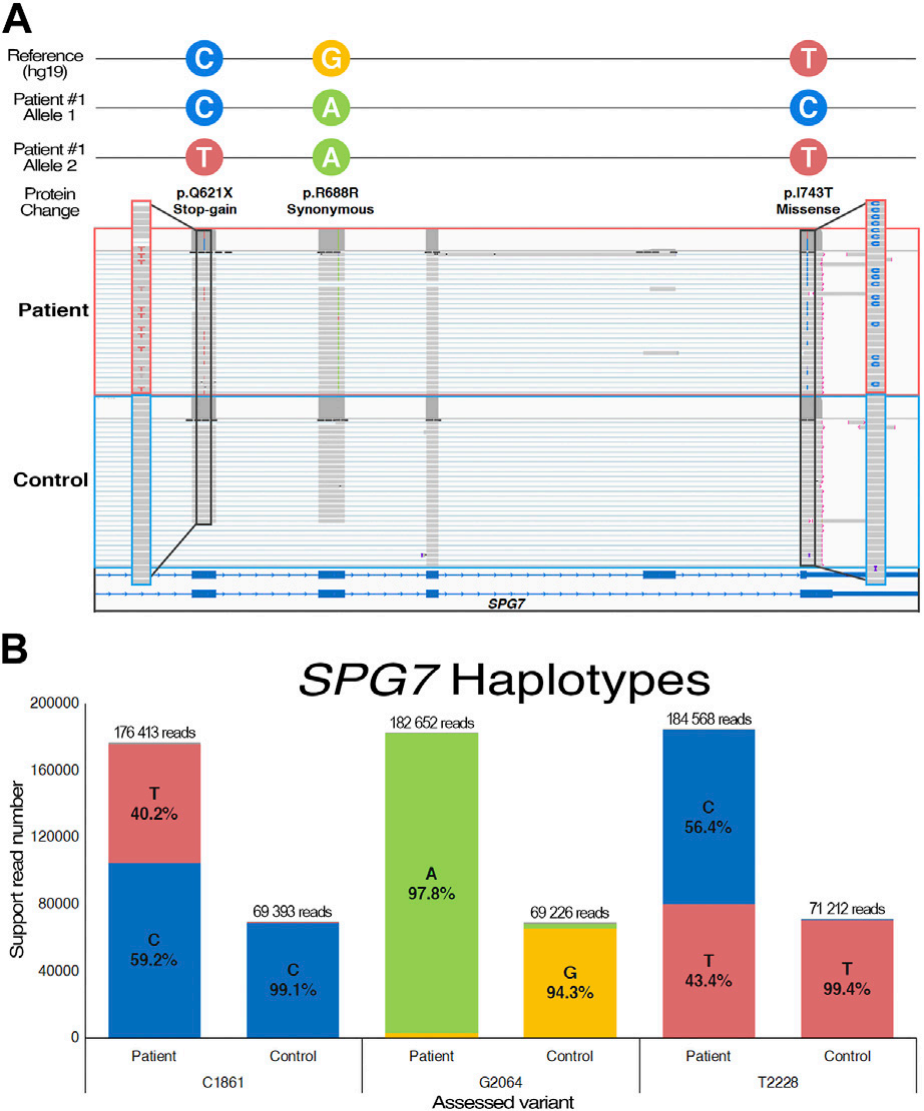
Long-read sequencing of the *SPG7* cDNA from individual #1. **(A)** Visual representation of the trans-configured variants on transcriptomic reads using the Integrative Genome Viewer. **(B)** Allele quantification utilizing a patient-specific synonymous variant (c.2064G>A) to confirm sample identity and base-calling accuracy. Control data was produced using comparable cDNA from a healthy sample.

Individual #1 is a male proband that was diagnosed with a progressive ataxia in his mid-fifties. However, there was noticeable but stable signs of speech and coordination difficulties since childhood. He is the only patient in which a potential acquired form of ataxia was not initially ruled out, as he recovered from a subdural hematoma after a car accident in his mid-twenties. Family history is limited, but two family members exhibited balance and speech issues, similar to those of individual #1, in the late stage of their lives. Clinical manifestations include strong episodes of imbalance with vertigo, ataxic gait, and nystagmus. Ophthalmoparesis as well as multiple auditory phenotypes such as progressive sensorineural hearing loss underscored the possibility of a mitochondrial disorder. Cerebellar atrophy was also observed by MRI. Genetic testing for both ataxia and mitochondrial relevant genes yielded mostly negative results (Table 1). Whole genome sequencing on the other hand revealed two distinct variants in the *SPG7* coding region (Supplementary Figure S1), a well-studied gene that causes an autosomal recessive spastic paraplegia as well as recessive cerebellar ataxias (Choquet et al., 2016a). The gene was prioritized due to the clear genotype-phenotype association, and the pathogenic potential of the identified variants (Supplementary Table S1). The missense (NM_003119: c.2228T>C/p.I743T) had previously been reported as likely pathogenic multiple times in ClinVar, while the nonsense variant (NM_003119: c.1861C>T/p.Q621*) has a near-zero allele frequency and has only recently been submitted as likely pathogenic. Both variants were also seen in the RNA-seq data, and were confirmed by Sanger sequencing for both the DNA and RNA (Supplementary Figure S1). In order to assess if both alleles were functionally affected by those variants, targeted LRS of complete *SPG7* mRNA amplicons confirmed that the two variants were *in trans* configuration (Figures 1A,B). Indeed, while reads from the control sample shows indistinguishable alleles containing no variants, reads from the patient, which can be confirmed by the homozygous synonymous variant (NM_003119: c.2064G>A/p.R688R), exhibit two clearly distinct alleles. Reads from individual #1 carry either the missense or the stop-gain variants, but never both. The identification of compound heterozygosity of two *SPG7* variants, previously reported as likely pathogenic according to ACMG guidelines (Richards et al., 2015), offers strong evidence of pathogenicity and sufficed to obtain a clinical diagnosis.

**TABLE 1:** Clinical presentation summary of ataxia cohort.

Cohort	ID #1	ID #2	ID #3	ID #4	ID #5	ID #6	ID #7	ID #8
Gender	M	F	M	M	M	M	F	M
Age (current)	71	84	79	83	76	45	49	86
Age (onset)	56	73	61	75	61	30	43	71
Ethnicity	French/Egypt	Italian	French Canadian	Czech	French Canadian	French Canadian	French Canadian	Polish
SARA score (at evaluation)	12.5 ^a^	7.5 ^a^	9.0 ^a^	18.0 ^a^	6.0 ^a^	1.0 ^a^	0 **^a^**	22.0
Episodes	Weekly	Infrequent	Daily	Monthly	3-4x/week	Daily	1-2x/month	Almost daily
Duration	Unknown	15 min	15 min–1 h	30 min	≤15 min	30 min–1 h	30 min	1–2 h
Triggers	Unknown	Unknown	Fatigue Exercises Coffee Hot drinks	Reading	Stress Fatigue	Stress Exercises Alcohol Sudden head movements	Stress Exercises	Fatigue Exercises
Progression	X	X	X	X	X	-^b^	X	X
Atrophy	Cerebellar Cervical	Cerebellar Bil. Temporal	-	-	-	Cervical (osteophytes)	-	Cerebellar (angioma)
Vertigo	X	-	-	X	-	-	X	-
Eye phenotypes	Smooth pursuit ↓NystagmusOphtalmoparesia	Smooth pursuit ↓ Nystagmus	Smooth pursuit ↓ Nystagmus	Smooth pursuit ↓ NystagmusSaccades (hyper)	Smooth pursuit ↓Nystagmus Saccades (hyper)	Smooth pursuit ↓ Nystagmus Ophtalmoparesia	Smooth pursuit ↓ Nystagmus	Smooth pursuit ↓ Nystagmus
Diplopia	Moderate	Mild	Mild	-	Mild	Mild	-	Mild
Dysarthria	Mild-Moderate	Moderate	Mild	Moderate	Mild	-	-	Mild-Moderate
Dysphagia	-	-	Mild	Mild	-	-	-	Mild
Dysmetria	Mild	Mild	Mild	Mild	Mild	-	-	Mild-Moderate
Reflexes	Hyper	Hyper	Hypo	Hypo	Hyper	-	Hyper	-
Impaired sensory system	Mild-severe auditory	Mild vibratory	Mild vibratory	Severe multiple	Moderate vibratory	-	-	Severe vibratory
Tremor	-	Intention	Mild action	-	Mild postural	-	-	-
Muscle weakness	-	-	X	Lower limb	-	-	-	X
Spasticity	Lower limb (Moderate)	-	-	-	Lower limb (Mild)	-	-	-
Treatment responses	-	-	Acetazolamide ± (stopped)	Acetazolamide ± (stopped)	-	-	Gabapentin ±	Acetazolamide – (stopped)
							Erenumab +	
Others	Babinski sign Dyslipidemia ↑ Vitamin E Hearing loss	Lypothymia Cataracts	Falls	Migraines Diabetes	Urinary dysfunction Peritonitis	Headaches Falls	Migraines Ocular pain Endometriosis Asthma	Arythmia Dyslipidemia
Family history	Sister (balance)	-	Father (similar)	-	Son (ID#6) Brother (similar)	Father (ID#5)	Sister (Spastic paraplegia)	-
Gene panel	Mitome-DG^e^ 163 genes (2014) Targeted *POLG SCA1-8* (2014)	NGS431^e^ ^,^ **^d^** 530 genes (2019) NGS420^g^ ^c^ 531 genes (2021)	NGS324^g^ 361 genes (2019) NGS431^g^ ^c^ 530 genes (2022)	Ataxia&DD^f^ 197 genes (2018)	Ataxia^h^ 150 genes (2018)	NDD14^f^ 108 genes (2018) Ataxia&DD^f^ 96 genes (2018)	NGS408^g^ 362 genes (2019)	NGS324.6^g^ 113 genes (2015) Targeted *KCNA1* *CACNA1A* (2015)
Negative hits	167 genes	528 genes	530 genes	191 genes	148 genes	200 genes	361 genes	113 genes
Positive hits(Report VUS)	*MTO1PCCB* *CYTB*	*MTOR* ^c^ *NAGLU* ^c^ *ELOVL4* ^c^	-	*RPGRIP1LSCYL1GBA2* *DMXL2ACO2PEX2*	*WFS1RPGRIP1L*	*WFS1*SPG7* *SYNE1* (2)*MTTP	*ITPR1*	*TTBK2* *SYNE1*

^a^ Recorded outside EA spells.

^b^ No recent progression.

c Ordered for diagnosis confirmation testing.

d Results unavailable.

e GeneDx™.

f CeGaT™.

g MNG, Laboratories™.

h Fulgent genetics.

### 3.2 Individual #2 *ELOVL4*

**FIGURE 2:**
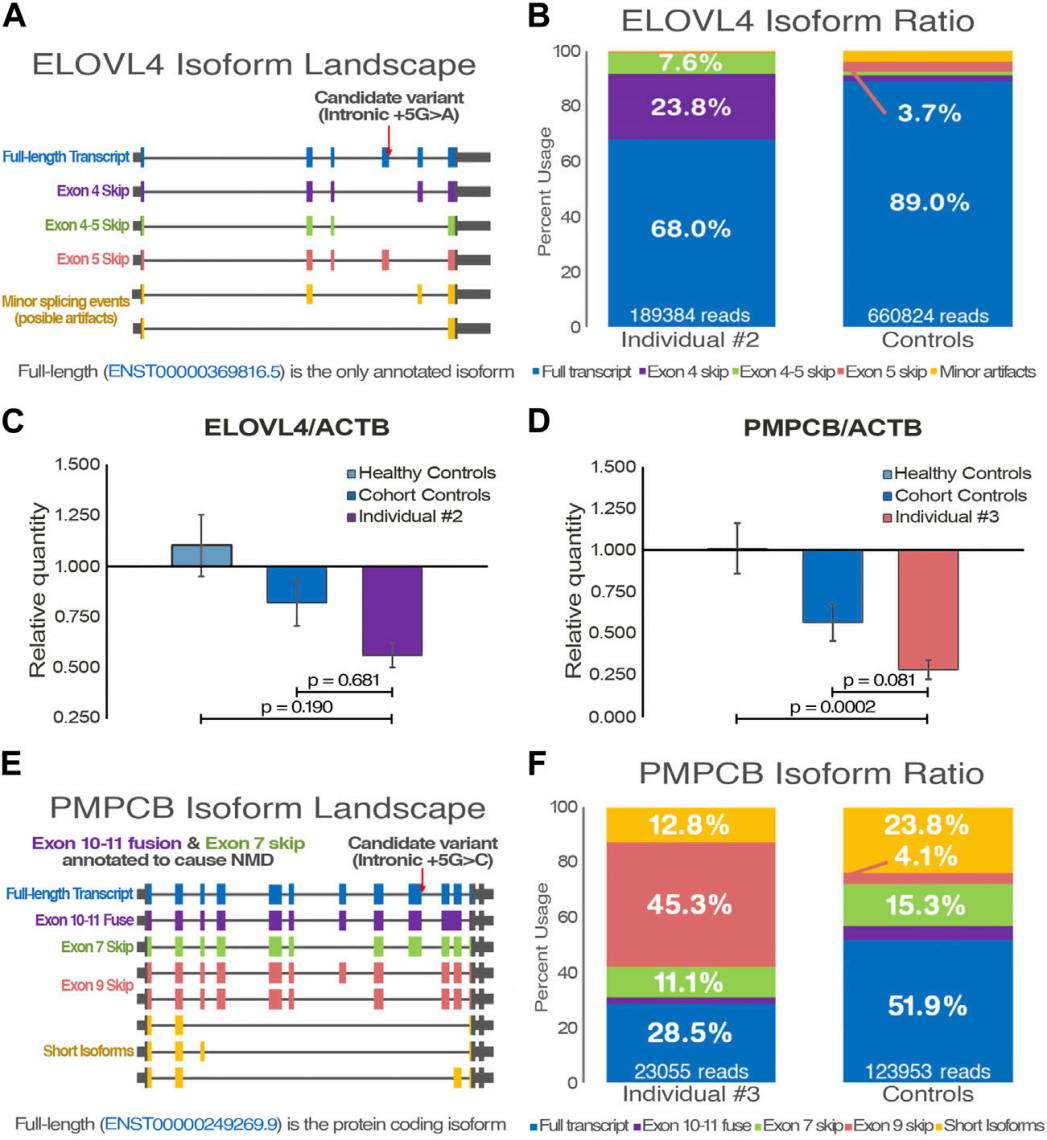
Functional transcriptomic validation of genes with suspected alternative splicing variants. **(A)** Visual representation of the *ELOVL4* isoform landscape following alignment on the hg19 reference genome. **(B)** FLAIR quantification of annotated and *de novo ELOVL4* transcripts from individual #2 and control samples (*n* = 3). **(C, D)** Gene expression quantification of qPCR data with an *ELOVL4* probe targeting exon 1–2 junction (NM_022726) and a *PMPCB* probe targeting exon 4–5 junction (NM_004279). *ACTB* is used for data normalization; Ataxia individuals are compared to both healthy (*n* = 3) and cohort (*n* = 2) controls. One-way ANOVA and Tukey’s *post hoc* multiple comparisons test were applied to calculate adjusted *p*-values and assess statistical significance. **(E)** Visual representation of the *PMPCB* isoform landscape following hg19 alignment. **(F)** FLAIR quantification of annotated and *de novo PMPCB* transcripts from individual #3 and control samples (*n* = 3).

First signs of disease in individual #2, a female proband without family history, manifested around age 73. Clinical presentation is predominantly characterized by episodes of dysarthria, but disease progression also led to ataxic gait as well as important episodes of nystagmus and diplopia. MRI revealed progressive atrophy of the cerebellum, but also a bilateral atrophy of temporal lobes. The latter prompted additional assessment of cognitive functions, which revealed that a mild cognitive impairment was in development (Hobson, 2015). Clinical genetic testing identified no pathogenic candidates (Table 1), but whole genome sequencing was able to detect an intronic variant in *ELOVL4* (NM_022726: c.541 + 5G>A), a gene known to cause autosomal dominant SCA34 (Supplementary Figure S1). Given the variant’s high pathogenicity score, null allele frequency in public databases, and its clear potential effect on the gene, it was prioritized for validation. While the variant has never been reported before, *in silico* predictions suggest it to be disease-causing through loss of exon 4 splice donor site (Supplementary Table S1). Coverage of *ELOVL4* in the RNA-seq was too low for variant calling, but it does appear in a few reads, and splice junctions suggested skipping of exon 4. After the variant was confirmed through Sanger sequencing (Supplementary Figure S1), PCR amplification of *ELOVL4* complete coding sequence enabled the analysis of its transcriptomic landscape through LRS (Figure 2A). The sequencing data confirmed that the intronic variant significantly caused skipping of exon 4, with an additional event that skipped both exon 4 and 5 (Figure 2B). While LRS isoform ratio analysis is relative, quantification of *ELOVL4* resulting mRNA levels was performed by qPCR (Figure 2C). The expression level is decreased by about 50% compared to healthy and diseased controls, suggesting nonsense-mediated decay of the alternatively spliced transcripts. The splicing alteration and subsequent decreased expression of *ELOVL4* observed in our patient demonstrate the deleterious effect of c.541 + 5G>A and is sufficient to classify this variant as pathogenic according to ACMG guidelines.

### 3.3 Individual #3 *PMPCB*

Individual #3 is a male proband who was diagnosed with progressive EA at age 61. Acute episodes are characterized mainly by gait ataxia, dysarthria, and nystagmus. Progressive ataxia baseline is accompanied by worsening of the upper and lower limbs muscle weakness after episodes. Mild action tremor was also noted, and inconsistent dysphagia has been observed (Table 1). While family history is incomplete, the father did exhibit signs of late-onset ataxia a few years prior to his death. Individual #3 also mentioned he has a few brothers with potential imbalance problems, which could suggest an autosomal dominant transmission, but clinical follow-up could not be achieved. Both WGS and RNA-seq identified an intronic variant in *PMPCB* (NM_004279: c.1154 + 5G>C) that is predicted to induce skipping of exon 9 (Supplementary Figure S1). Once more, analysis led to prioritization of the variant due to high scores from pathogenicity prediction tools, low allele frequency, and literature supporting a role in cerebellar functions (Supplementary Table S1). Transcriptomic data, despite a relatively low coverage, does support this potential alternative transcript. DNA Sanger sequencing confirmed the variant before further assessment by qPCR and LRS (Supplementary Figure S1). A significant decrease of over 50% of the gene expression in qPCR data suggests that the affected allele is eliminated through nonsense-mediated decay (Figure 2D). Amplification of the most commonly expressed annotated transcript by targeting shared exons yielded a near complete transcriptomic landscape of *PMPCB* (Figure 2E). LRS data once again strongly support the loss of exon 9 in multiple transcripts (Figure 2F), and provides a probable explanation for the nonsense-mediated decay. While they are not regarded as the main disease-causing variants, it is important to note that the WGS also uncovered two variants in *CACNA1G*, albeit neither are considered likely pathogenic due to their considerable allele frequencies.

### 3.4 Individual #4 *ATXN2*

**FIGURE 3:**
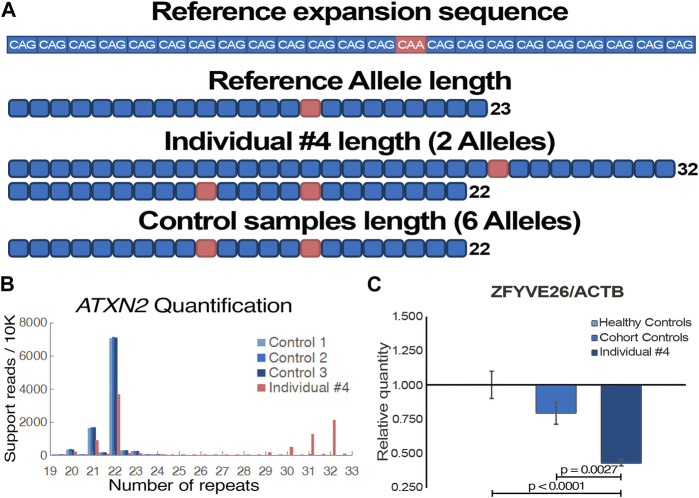
Functional validation of individual #4 pathogenic genomic alterations. **(A)** Visual representation of *ATXN2* trinucleotide repeat expansion sequence and length from individual #4 and control samples (*n* = 3) following targeted long-read sequencing. **(B)** Tandem-genotypes quantification of the samples’ *ATXN2* trinucleotide repeat lengths. Supporting read numbers are scaled per 10,000 reads to facilitate visualization of samples alleles. **(C)** Gene expression quantification of qPCR data with a *ZFYVE26* probe targeting exon 16–17 junction (NM_015346). *ACTB* is used for data normalization; Individual #4 is compared to both healthy (*n* = 3) and cohort (*n* = 2) controls. One-way ANOVA and Tukey’s *post hoc* multiple comparisons test were applied to calculate adjusted *p*-values and assess statistical significance.

Acute episodes of dysarthria, vertigo and ataxia first happened when individual #4 was 75 years old. Disease progression was observed despite a positive response to acetazolamide, as episodes are more frequent and now include nystagmus, migraines, as well as muscle weakness and paresthesia of the lower limbs. The condition’s progression into a chronic state remains uncertain but plausible. Clinical genetic testing, which did not cover repeat expansions, included 197 ataxia-related genes and identified no variants that could explain individual #4 phenotypes (Table 1). Using WGS data (Supplementary Figure S1), the Expansion Hunter tool predicted a repeat expansion of *ATXN2* exon 1 CAG microsatellite, which was prioritized due to the clear association to cerebellar ataxia, and a lack of other clear causative variants. Using LRS on *ATXN2* DNA amplicons, alleles of 22 and 32 repeats were detected in individual #4 (Figures 3A,B), positioning him directly on the SCA2 threshold of variable penetrance and delayed onset (Almaguer-Mederos et al., 2010). Interestingly, both sequencing datasets also identified a heterozygous nonsense variant in *ZFYVE26* (NM_015346: c.3022C>T/p.R1008*), which has been reported as pathogenic (Supplementary Table S1). For this reason, we hypothesized that the variant could potentially act as a disease modifier of the patient’s phenotype. The variant was confirmed by Sanger sequencing (Supplementary Figure S1), and a strong decrease in the gene expression was observed by qPCR (Figure 3C), suggesting nonsense-mediated decay of the affected transcript once more.

### 3.5 Individual #5 and #6

Individual #5 is a male proband who first consulted for episodes of gait instability around age 61, but had short transitory episodes of diplopia in his mid-thirties. Additional clinical manifestations following disease progression include dysarthria, nystagmus, hyperreflexia and mild postural tremor. Family history revealed that one of five siblings also exhibits gait imbalance and mild tremors akin to those of the patient. He is also the father of individual #6, who exhibits a similar early ocular phenotype, as well as an unaffected daughter. Individual #6 disease onset was around age 30, which could be due to a mild traumatic brain injury that occurred 10 years prior, but an anticipation effect was considered. As expected from his father’s medical history, there has been no disease progression yet, and the main feature of episodes is diplopia. Neither patient exhibit cerebellar or cervical atrophy. Genetic testing, which covered over 200 ataxia genes, identified only one common VUS in *WFS1*, and no clear disease-causing *de novo* variants (Table 1). WGS and RNA-seq did not detect pathogenic variants in known ataxia genes, but did identify shared VUS in two genes that we are currently investigating as potentially relevant to cerebellar functions. VUS unique to a single patient are also being considered in case of distinct pathologies.

### 3.6 Individual #7 and #8

Individual #7 is a female proband with a disease onset at age 43. Acute episodes of vertigo, nystagmus, and migraines have progressed in intensity over time, but few new clinical manifestations have been observed. Ataxia is mainly observed in the lower limbs, and there is no cerebellar atrophy at this time. Lack of family history suggests a *de novo* pathology, albeit her mother has history of epilepsy, but clinical genetic testing yielded no candidate variants, as a single *ITPR1* VUS was found (Table 1). Individual #8 is a male proband who first exhibited episodic signs of cerebellar dysfunction around the age of 71. Symptoms were typical: ataxic gait, nystagmus, dysarthria, as well as cerebellar atrophy which was revealed by CT-scan. Although current clinical presentation appears to fit EA, the increasing frequency and duration of episodes implies that progression into a chronic state cannot be ruled out. Gene panels for 115 genes failed to identify conclusive pathogenic candidates, revealing VUS only in *TTBK2* and *SYNE1* (Table 1). WGS and RNA-seq variant analysis also did not identify obvious candidates, but variants in genes with brain-restricted expression are currently being investigated as potential novel causes of the individual’s phenotypes.

## 4 Discussion

The results of this study highlight the relevance of multi-omics approaches in genetic investigations deemed complex following a failure from first-line clinical testing, or even in contexts where the suspected pathology is not clear. Although phenotype-driven genomic investigations are generally efficient diagnostic strategies, certain Mendelian disorders pose additional challenges that render the option less viable, such as a late onset of phenotypes, high clinical heterogeneity, variable penetrance, and unpredictable disease progression (Pengelly et al., 2017). Our cohort of individuals with late-onset progressive EA-like disorders presented several of these challenges, as most had limited family history and showed no identifiable causative variants in standard gene panels testing (Table 1). In this study, the rationale behind the combination of WGS and RNA-seq lies in the knowledge that known pathogenic variants are no longer expected, albeit some could have been missed during clinical probing. In an exploratory context, the WGS technology offers the most comprehensive overview of a patient’s genomic state (Koboldt, 2020). While there is currently limited information available regarding the biological relevance of non-coding regions, which represent 98% of the genome, it is thought that up to 82% of it could have regulatory functions (Gloss and Dinger, 2018). Although most non-coding variants might have less direct pathogenic impacts, they undoubtedly contribute to the relatively modest clinical diagnostic success rates, around 25%–50%, obtained from clinical methods focusing primarily on coding regions (Scacheri and Scacheri, 2015; Ellingford et al., 2022). Despite the advancements in algorithms that predict functional impacts of whole genome variants, such as SpliceAI which demonstrates impressive performances and will likely improve further along with new literature (Jaganathan et al., 2019), the interpretation of non-coding variants as well as VUS remains the significant limitation of WGS. As such, RNA-seq offers a complementary functional insight on the transcriptomic states of genes carrying those variants (Yepez et al., 2022; Lunke et al., 2023). This dynamic view of gene expression and splicing in relation to genomic alterations is highly valuable to variant classification and prioritization. Therefore, we believe the approach offers the best odds at reaching a molecular diagnostic in patients with Mendelian disorders deemed complex.

Identification of non-synonymous mutations in the *SPG7* gene of individual #1 was a surprising finding, as they technically should have been caught by gene panel testing. Unsuccessful identification of causal variants by first-line clinical testing can stem from technical or bioinformatics issues (Murray et al., 2022), but in this case was simply due to the fact that *SPG7* was not included on the selected ataxia and mitochondrial genes panel at the time of testing (Table 1). This underscores the dynamic nature of VUS literature, but also that leveraging more complete sequencing approaches enables retrospective analysis of emerging pathogenic gene associations. The *SPG7* gene encodes a mitochondrial metalloprotease, and its dysfunction is a common cause of an adult form of recessive spastic paraplegia and cerebellar ataxias (Pfeffer et al., 2015). The patient’s observed phenotypes are consistent with *SPG7*-related pathologies, which encompass both complicated and pure forms, with some carriers even receiving a diagnosis of spastic ataxia. It is important to note that while the individual made a near complete recovery from the subdural hematoma he suffered in his early adulthood, it is not possible to conclude on whether it influenced the clinical phenotypic presentation or not. However, both variants of individual #1 have been reported as likely pathogenic at least once: the missense variant (NM_003119: c.2228T>C/p.I743T) causes a change in amino acid within the peptidase domain, which is predicted to have deleterious effects *in silico* (Supplementary Table S1), while the stop-gain variant (NM_003119: c.1861C>T/p.Q621*) almost certainly causes loss-of-function through protein truncation and nonsense-mediated decay (Choquet et al., 2016a). The usage of LRS to clearly demonstrate the trans configuration of the two pathogenic variants is sufficient evidence to obtain a clear diagnosis of recessive spastic ataxia with episodic manifestations. Although persistence of symptoms is suspected to arise from future disease progression, the episodic nature of phenotypes was a prominent feature of clinical presentation. The results also highlighted a strength of the technology, which is the ability to perform accurate genome-wide allele phasing prior to variant calling. This feature is valuable to the identification of compound heterozygosity, especially in late-onset cases where parents are unavailable for segregation analysis (Akbari et al., 2021).

Intronic variants offer compelling illustrations of the benefits of combining WGS and RNA-seq. The *ELOVL4* variant (NM_022726: c.541 + 5G>A) was called by GATK in the genomic dataset, but the coverage was seemingly too low to be detected in the RNA-seq data. However, a manual examination of the transcriptomic landscape of *ELOVL4* using Integrated Genome Viewer (IGV) revealed that a few reads did support the *in silico* prediction of exon 4 splice donor loss resulting in skipping (Supplementary Table S1). The observation greatly assisted in prioritizing this variant, as potential splicing events frequently have otherwise unremarkable pathogenic scores compared to non-synonymous variants called in WGS (Rentzsch et al., 2021). Once more, leveraging LRS as a functional validation tool, we performed targeted sequencing of *ELOVL4* transcripts in individual #2, which confirmed a clear shift in isoform ratio (Figure 2B). Interestingly, our findings revealed that exon 5 exhibited a natural predisposition to splice defects, resulting in approximately 25% of the patient’s alternatively spliced transcript skipping both exon 4 and 5 instead of only the predicted exon 4. Moreover, individual #2 presents a near 10-fold increase of exon 4 skipping, which represents around 30% of all reads and supports the hypothesis that alternatively spliced transcripts are subject to nonsense-mediated decay. The decrease in mRNA expression also corroborates this hypothesis (Figure 2C). *ELOVL4* encodes an enzyme that is involved in fatty acids biosynthesis, and is already associated to the dominant form of SCA34 (Gyening et al., 2023). Both abnormal exon-skipping events observed in individual #2 also induce a frameshift, leading to major structural changes at the protein level which ultimately results in a complete loss of native catalytic functions. The rationale we posit for the prominent episodic nature of the patient’s phenotypes is that the biological role of *ELOVL4* is dosage-dependent: in baseline circumstances, cells compensate the enzyme function with their healthy allele, effectively distinguishing and degrading the alternatively spliced transcripts in parallel (Fahrner and Bjornsson, 2014). However, events that lead to increased enzymatic processing of fatty acids would act as EA triggers. This hypothesis could also provide an explanation for the absence of erythrokeratodermia, a frequently observed but not obligatory persistent phenotype of SCA34 (Nishide et al., 2023).

Individual #3 presents a very similar case, except the *PMPCB* variant (c.1154 + 5G>C) was called in both datasets, albeit with lower pathogenicity prediction scores. Like the previous case, IGV examination supported the *in silico* prediction of splice donor loss and exon 9 skipping. Intriguingly, LRS revealed that while only the full-length transcript (ENST00000249269.9) of *PMPCB* is expected to be functional (Vogtle et al., 2018), minor isoforms carrying alternative splicing such as exon 7 skipping or a fuse of exon 10 and 11 accounted for nearly 50% of all reads in control samples (Figure 2F). Nevertheless, the isoform ratio analysis still reveals a clear shift for individual #3, whose data suggest that all transcripts from the variant-carrying allele are predictably missing exon 9, even as other alternative splicing events also occur. While transcripts missing exon 9 account for 45% of reads in the LRS data, the 2-fold decrease observed in mRNA expression data (Figure 2D), as well as in the RNA-seq (Supplementary Table S1), suggests nonsense-mediated decay of affected allele may still be happening. The *PMPCB* gene encodes for the catalytic subunit of the essential mitochondrial processing protease (MPP), and the bi-allelic loss-of-function has been shown to cause multiple mitochondrial dysfunctions syndrome-6 (MMDS6), a severe and early-onset neurodegenerative disorder (Vogtle et al., 2018). While individual #3 phenotypes are less severe and more tardive, there is a strong overlap in the type of clinical manifestation, and a milder autosomal dominant presentation of a known harsh recessive disorder has been observed numerous times in rare disorders literature (Tetreault et al., 2015). As loss of exon 9 results in the loss of a significant part of the MPP-β, specifically two α-helix and two β-sheets towards the inside of the protein (UniProt, 2023), *in silico* tools unanimously predict misfolding and complete loss-of-function of the resulting peptidase (Supplementary Table S1). As MPP-β is essential for the processing of proteins associated with neurodegeneration, namely FXN and PINK1 (Schmucker et al., 2008; Greene et al., 2012), it is highly probable its alteration negatively impacts cerebellar functions, and could even explain the mild action tremors observed in individual #3. Therefore, we propose a similar dosage-dependent pathogenic effect of the variant, where a single healthy *PMPCB* allele is enough for baseline functions, but increased mitochondrial activity causes dysfunctions that are similar to those seen in MMDS6, albeit on a smaller scale. While further functional investigation and additional patients will help understand the role of *PMPCB* in ataxic phenotype, we posit that ataxia gene panels would benefit from the inclusion of the gene in a similar fashion to its partner, *PMPCA* (Choquet et al., 2016b). Given that the two are functional partners, it is reasonable to hypothesize disruptions of *PMPCB* functions, thereby hindering MPP mitochondrial role, could cause a very similar condition (Kunova et al., 2022).

While RNA-seq provided valuable insights into potential pathogenic alternative splicing and changes in expression associated with candidate variants, we firmly believe that the additional information offered by LRS makes it a formidable asset for the functional validation of VUS. As long-read technology providers approach the capability of offering full-length whole-transcriptome data with unique molecular identifiers, accurate assessment of differential expression at the isoform level becomes feasible. This extent of data resolution holds immense potential, especially as pathogenic effects of variants can be driven in isoform-specific manners (Rudell et al., 2019), and compensatory mechanisms underlying discrepancies between gene expression and functional impact could be more readily identified. Although the RNA-seq and qPCR data did not demonstrate statistically significant, albeit substantial, decreases in *ELOVL4* levels for the patient, examination of the LRS results clearly revealed a significant decrease in functional transcripts (Figure 2B). Hence, it enables the hypothesis that there is compensatory expression from the healthy allele to partially rescue the function, which could help elucidate the episodic nature of the phenotypes. The implementation of phased calling features, currently being optimized for long-read platforms, is poised to reveal numerous additional instances of this phenomenon (Akbari et al., 2021). Moreover, LRS is currently being utilized to construct a highly accurate and cell-specific human transcriptome that does not rely on isoform prediction, directly enhancing the ability to detect abnormal splicing and isoform-specific differential expression (Reese et al., 2023). The decreasing cost per sample and the steady improvement in LRS base calling accuracy have been effectively mitigating current limitations of the technology, underscoring its growing potential for clinical genetics.

In a similar manner, increased length of native genomic reads, as well as the ability to detect nucleotide modifications such as methylation, give a slight edge to the potential of LRS (Akbari et al., 2021). One of the shortcomings of traditional WGS is the difficulty of detecting structural variants larger than a few hundred base pairs. As standard sequencing read length is too short to confidently anchor the alternative sequences to clear break points in the genome, most structural variant calling tools for short reads derive information from atypical read-splitting, inconsistent read pairs, and abnormally low read depth for the region (Kosugi et al., 2019). Assembly approaches, which aim to make contigs spanning as far as possible from the break points, along with combinatory tools, generally perform best as they more accurately detect abnormal events. LRS approaches provide a significant advantage in identifying large structural variants, as every read generated by this technology contains information comparable to a contig. Similarly, repetitive regions have long represented a fundamental challenge for variant calling of sequencing. As such, Expansion Hunter is one of the few tools that can be used on short-read data to call repeat expansions, a common cause of neurodegenerative disorders (Dolzhenko et al., 2020). Moreover, it still just offers a broad prediction of the microsatellite expansion length. Therefore, LRS offers a unique opportunity to observe full-length pathogenic repeat expansions anchored to the reference genome. As tools are being developed and optimized, it is feasible to detect and quantify the number of short-tandem repeats in each sample, which will likely lead to the detection of numerous new repeat expansion disorders (Pellerin et al., 2023). Individual #4 offered a neat example of the clinical potential of targeted repeat expansion amplification, as precise repeat quantification could be used in multiple ataxia-related disorder testing. Combined with phased-alignment, it is possible to obtain a more accurate quantification of the repeat number on each allele, revealing here that the patient is directly on the threshold of variable penetrance (Laffita-Mesa et al., 2012). It is interesting to observe the characteristic loss of one of the CAA interruptions, which is believed to influence functional transcripts by disrupting the formation of CAG hairpins. While mechanisms of clinical variability in *ATXN2* expansions remains unclear, it is hypothesized that these CAG hairpins contribute to pathogenicity by altering protein interactions, as well as mRNA localization and processing in a manner that may be tissue-specific (Marti, 2016; Fournier et al., 2018). Alleles of ≥31 CAG repeats have also been shown to be risk alleles for amyotrophic lateral sclerosis with or without frontotemporal dementia, suggesting *ATXN2* pathogenic mechanisms are complex and dynamic (Glass et al., 2022), and that other variants or genes may be implicated in the variable pathogenesis. Although rare, alleles of 32 and 33 repeats have been described as significantly delaying the age of onset (Daoud et al., 2011; Neuenschwander et al., 2014). This finding is consistent with the episodic and late-onset nature of the phenotypes compared to typical SCA2 patients with large *ATXN2* expansions (Figueroa et al., 2017).

The concept of genetic modifiers, although predominantly explored in the context of genome-wide association studies, holds substantial relevance to the field of clinical genetics, offering potential explanations for the high phenotypic heterogeneity observed in complex genetic disorders (Rahit and Tarailo-Graovac, 2020). The suggested impact of disease modifiers encompasses not only the diversity and severity of observed symptoms, but could potentially influence the age of onset as well as the rate of disease progression (Li et al., 2021). Individual #4 presents an interesting example of the potential of a genetic modifier impacting disease phenotype. The very significant decrease in gene expression (Figure 3C), which supports *in silico* predictions of a non-functional protein resulting from the truncating variant (Supplementary Table S1), is partly compelling evidence that *ZFYVE26* is implicated in the patient’s phenotype. Furthermore, the patient clearly present clinical features associated with both *ATXN2* and *ZFYVE26* related disorders. Nevertheless, additional validation is required to confirm a genetic modifier role, as related spastic paraplegias are generally recessive (Saffari et al., 2023). Additional SCA2 patients carrying heterozygous pathogenic variants in *ZFYVE26* or modeling of both variants could help confirm this hypothesis. In addition, having access to isoform level quantification as well as epigenetic data directly from the combined sequencing results may have provided the additional information required to confidently make the assessment on the role of *ZFYVE26*. Indeed, the growing capability to evaluate epigenetic markers using LRS data is highly interesting in the context of predicting the functional effects of VUS, and particularly non-coding variants (Frydas et al., 2022). There are several examples in the literature describing genetic modifiers acting in synergy with the main genetic variant to influence phenotypes. Illustratively, a *SQSTM1* variant known to cause a multisystem proteinopathy through stress granule impairment was enhanced synergistically by a variant in *TIA1*, gene annotated to cause a distinct myopathy (Lee et al., 2018). This concept has consequently been explored to elucidate the marked clinical diversity observed in SCAs (Wang et al., 2019). As clinical genetic testing decisions mostly rely on the standard phenotype-driven approach, improving the classification of genotype-phenotype relationships can aid healthcare providers in obtaining accurate diagnoses through first-line testing. Additionally, it has been proposed that modifier variants can influence, whether positively or negatively, the efficacy of therapeutic interventions, akin to haplotype-specific drug responses (Ahsan et al., 2020). This emphasizes the importance of identifying and understanding disease modifiers, as it can significantly impact multiple facets of clinical genetics and pharmacogenomics, aligning with the goal of achieving personalized medicine.

Despite being more time and resource consuming, extensive exploratory research on patients with suspected complex genetic causes are essential to the broader improvement of clinical genetics. Reclassification of VUS following functional assessments, as well as identification of novel causative genes and molecular mechanisms of pathogenesis greatly assist future variant prioritization during clinical genetic testing. Despite having a relatively small cohort, since atypical EA are highly rare and complex disorders, the combination of WGS and RNA-seq enabled the identification of pathogenic variants in four individuals (50%). Three of them should improve genotype-phenotype classification of known ataxia genes, as the variants seemingly cause atypical episodic clinical manifestations that otherwise resemble their classic disorder counterpart. Although *PMPCB* was previously only associated to a severe recessive mitochondrial disorder with ataxia phenotypes, we propose that loss-of-function of a single allele can lead to a milder late-onset dominant neurodegenerative phenotype. Our findings provide further evidence to broaden the genetic analysis to other genes, such as those directly or indirectly affecting ion channels or mitochondrial function, in patients presenting EA-like phenotypes or typical EA who do not carry disease variants in the respective genes (Giunti et al., 2020). Additionally, we are optimistic that the candidate variants identified in genes that are not yet associated to neurodegenerative disorders for the four remaining participants may lead to an improved diagnostic yield. While related literature is limited, elements such as suspected function, expression patterns, interaction partners, and preliminary results all support a potential role in cerebellar functions. Novel associations that may result from the on-going extensive tissue-appropriate modeling and functional validation would further support the efficiency of the approach in clinical contexts. While satisfied with the results obtained through the presented multi-omics approach, we believe an LRS-centered adaptation could perform even better, as part of the functional validation could be accomplished with the initial sequencing data. The proposed approach would offer genomic, transcriptomic, and epigenetic information in the most optimal resolution currently available, increasing the likelihood of attaining a molecular diagnosis. Consequently, we hope its application will benefit a broader spectrum of Mendelian disorders. Ultimately, the goal is to help define an optimal workflow of genetic testing, leveraging the benefits of continually evolving molecular technologies. Despite the perceived high cost of extensive second-line genetic testing, its value lies in the profound knowledge it offers. Moreover, it has the potential to reduce future care management costs through the development of personalized therapeutic strategies, thus maximizing the overall benefits for the patient.

## Data Availability

The data presented in this study are deposited in the NCBI BioProject repository, accession number PRJNA1014106, https://www.ncbi.nlm.nih.gov/bioproject/1014106. Variant reports have also been deposited in NCBI ClinVar repositories. The names of the repositories and accession numbers can be found below: https://www.ncbi.nlm.nih.gov/clinvar/variation/215218/, VCV000215218.46 (SPG7-missense). https://www.ncbi.nlm.nih.gov/clinvar/variation/1526041/, VCV001526041.4 (SPG7-nonsense). https://www.ncbi.nlm.nih.gov/clinvar/variation/2580357/, VCV002580357.1 (ELOVL4). https://www.ncbi.nlm.nih.gov/clinvar/variation/2580358/, VCV002580358.1 (PMPCB). https://www.ncbi.nlm.nih.gov/clinvar/variation/948359/, VCV000948359.5 (ZFYVE26).
