# Supplementary material for: Integration of multi-omics technologies for molecular diagnosis in ataxia patients
Source: Front Genet. 2024 Jan 4;14:1304711. doi: 10.3389/fgene.2023.1304711 (PMC10794629; doi:10.3389/fgene.2023.1304711)
Supplement: Supplementary file 1 [file DataSheet1.docx]

Supplementary Material

Integration of Multi-Omics Technologies for Molecular Diagnosis in Ataxia Patients

Sebastien Audet^1,2*^, Valerie Triassi^1^, Myriam Gelinas^3^, Nab Legault-Cadieux^1,2^, Vincent Ferraro^3^, Antoine Duquette^1,2,4,5^, Martine Tetreault^1,2^

*** Correspondence:** Corresponding Author: sebastien.audet.4@umontreal.ca

# Supplementary Figures and Tables

## Supplementary Figures

**Supplementary Figure 1. (A-F)** Integrative genome viewer (IGV) screenshots of whole-genome sequencing data from candidate variants samples (top half) and a control sample (bottom half). **(G-K)** Chromatograms from Sanger sequencing validation of candidate variants. **(A, G)** Heterozygous C›T stop-gain variant from individual #1 at coding position c.1861 of *SPG7* (NM_003119). **(B, H)** Heterozygous T›C missense variant from individual #1 at coding position c.2228 of *SPG7* (NM_003119). **(C, I)** Heterozygous intronic G›A splicing variant from individual #2 at non-coding position c.541+5 of *ELOVL4* (NM_022726). **(D, J)** Heterozygous intronic G›C splicing variant from individual #3 at non-coding position c.1154+5 of *PMPCB* (NM_004279). **(E, K)** Heterozygous C›T stop-gain variant from individual #4 at coding position c.3022 of *ZFYVE26* (NM_015346). **(F)** *ATXN2* exon 1 polyglutamine region (NM_002973) from individual #4 and a healthy control.


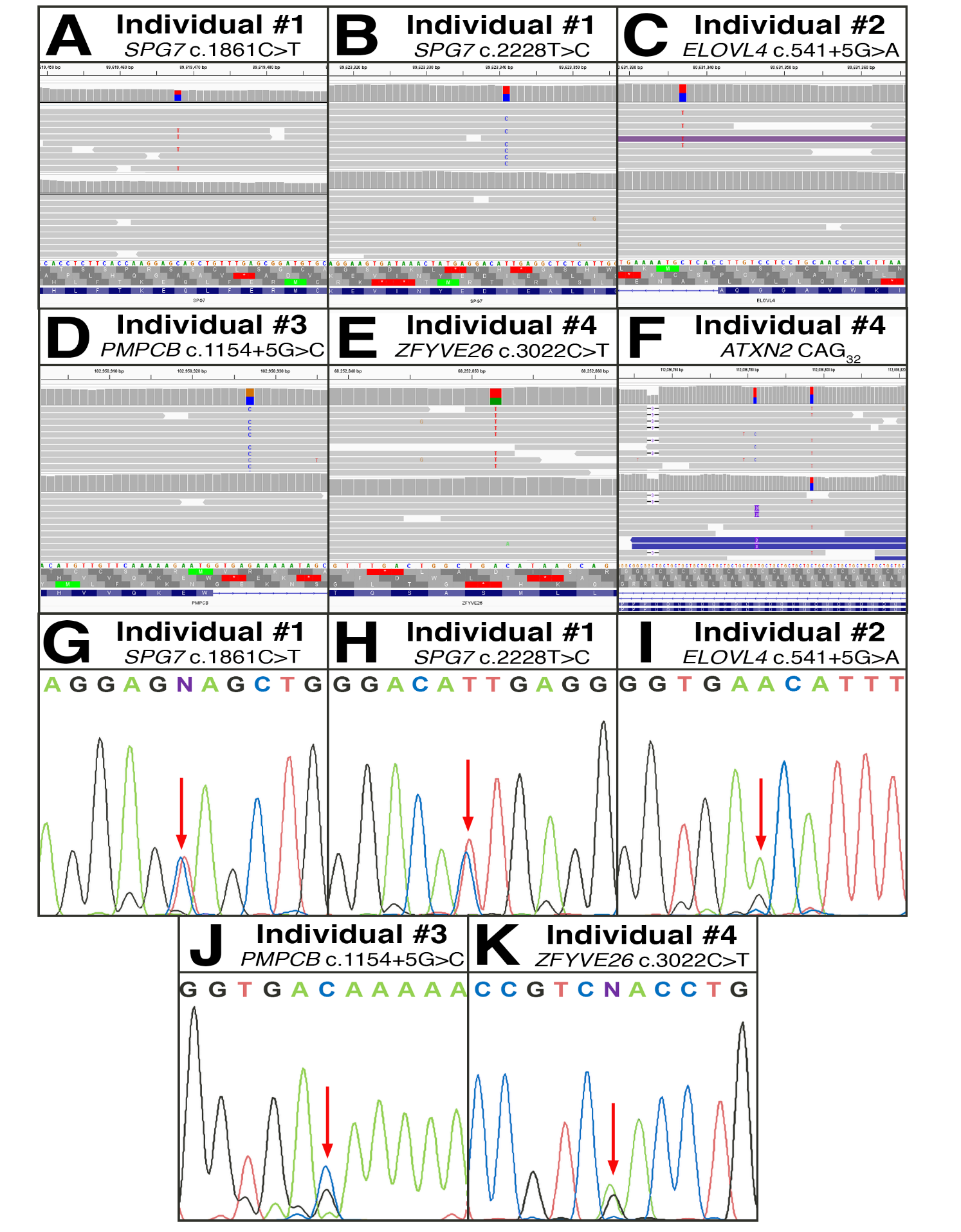


## Supplementary Tables

Supplementary Table 1. Summarized characteristics of candidate variants

| ID | Variants | Annotation | *In silico* | Associated disorders | Primers |
| --- | --- | --- | --- | --- | --- |
| **Indiv. #1**  **Seq Tag:** MT-0012 | ***SPG7*** (NM_003119)  c.1861C>T p.Q621X (Stop-gain)  c.2228T>C p.I743T (Missense) | CADD score: 39 gnomAD AF: 0  CADD score: 25.4 gnomAD AF: 4.95x10^-5^ | Log2FC: 0.060 (ns)  Patho-like: c.1861 98.5% c.2228 78% | Spastic paraplegia 7 (AR) | DNA1_F: TGCCTTCCTGCTTTGAGACG  DNA1_R: TGCACTGGAACAGAAGGAGTC DNA2_F: TGAGGTTGAGATGGGGGTGA  DNA2_R: TCGCCCAAGTCCTGTTTCTC cDNA/LRS_F: AGAACAGAAAGTGGTTGCGT  cDNA/LRS_R: CCCAAGTCCTGTTTCTCCCT |
| **Indiv. #2**  **Seq Tag:** MT-0009 | ***ELOVL4*** (NM_022726)  c.541+5G>A (Splicing) | CADD Score: 19.4 gnomAD AF: 0 | Log2FC: 0.213 (ns)  Patho-like: 84% | Spinocerebellar ataxia 34 (AD) | DNA_F: AAGGAGTTGAGTATTTGGACACA  DNA_R: CAAAGTCCTAGGTTCTCATTGCT  cDNA_F: TCTGATGCAGTCTCCTTGGC  cDNA_R: GGAAGGGGCAGTCAGTGTAA LRS_F: GGAGCCGGGTAGTGTCCTAA LRS_R: TCATGGCTGTTTTTCCAGCTTT |
| **Indiv. #3**  **Seq Tag:** MT-0010 | ***PMPCB*** (NM_004279)  c.1154+5G>C (Splicing) | CADD Score: 16.7 gnomAD AF: 4.91x10^-5^ | Log2FC: -0.576  (p = 1.4e^-9^)  Patho-like: 89% | Multiple mitochondrial dysfunction syndrome 6 (AR) | DNA_F: AAGCGTATGTAGCCAAGAGTCC  DNA_R: GGAAAAACCAACTGCAACCTTTG  cDNA_F: CTCTGCCTCCCTGCAAATTC  cDNA_R: GGGACCAACAGCAGCAATAG LRS_F: TCGAGTGGTGTTGTCATCCG  LRS_R: GCAGCAATAGCTGGACTCCT |
| **Indiv. #4**  **Seq Tag:** MT-0013 | ***ZFYVE26*** (NM_015346)  c.3022G>A p.R1008X (Stop-gain) | CADD Score: 42 gnomAD AF: 0 | Log2FC: -0.152 (ns)  Patho-like: 100% | Spastic paraplegia 15 (AR) | DNA_F: TGAAGGCAGTACCAAGGCAA  DNA_R: GCTGACCTAATGTTCCAAGTCC  cDNA_F: GAACTCAGATGCGGGTAGCA  cDNA_R: GGCAACACAGTCCTCGCTTA |
| **Indiv. #4**  **Seq Tag:** MT-0013 | ***ATXN2*** (NM_002973)  CAG_32-31_ (Expansion) | CADD Score: - gnomAD AF: - | Log2FC: -0.170 (ns)  Patho-like: - | Spinocerebellar ataxia 2 (AD) | DNA_F: CTTCGTCGTCCTCCTTCTCC  DNA_R: TCCCTCCATCTTGACCGC  LRS_F: CAAACTAGGTCCCCGCAGAA  LRS_R: ATTTAAGGAGGACGCCGGTC |

AF: Allele frequency; AD/AR: Autosomal dominant/recessive; Log2FC: Log2 fold change;
Patho-like: Pathogenicity likeliness score (tree mode) from Mutation Taster.
Log2FC analysis performed with DESeq2 v.1.6.2 with other samples as diseased-controls (n = 8).
